# Laser Fabrication and Comparative Study of Planoconcave and Planoconvex Microlenses on Fused Silica and Sapphire

**DOI:** 10.3390/mi16060608

**Published:** 2025-05-23

**Authors:** Narayana R. Gottumukkala, Caleb Barnes, Mool C. Gupta

**Affiliations:** Department of Electrical and Computer Engineering, University of Virginia, Charlottesville, VA 22903, USA; nrgottumukkala@gmail.com (N.R.G.); cdb8sg@virginia.edu (C.B.)

**Keywords:** laser, micro fabrication, lens, planar lens

## Abstract

We report on fabricating planoconcave lenses using a picosecond 355 nm wavelength laser and a CO_2_ laser. We also report the fabrication of the planoconvex microlens array on fused silica by patterned micromachining using a picosecond laser and reshaping using a CO_2_ laser. We report results on the surface morphology, profile, roughness, optical transmission efficiency, and laser beam profile of transmitted light passing through the microlens. We demonstrate laser fabrication of planoconcave lenses on infrared transmitting material sapphire. Furthermore, we present the results of an experimental and simulation comparative performance study of planoconcave microlenses obtained by individual picosecond and CO_2_ lasers.

## 1. Introduction

As imaging systems are becoming extremely miniaturized, the need for high-efficiency micro-optical elements is growing. Microlens arrays consist of many miniature lenses, like an insect’s compound eye. They have lower image resolution and sensitivity than macro lenses, so they are more suitable for fiber coupling [1], light beam homogenizers [2], laser beam shaping [3,4], light field cameras [5], LED light management [6], 3D integral imaging [7,8], and increasing efficiency in photovoltaics [9,10]. Recently, high-power lasers have been used for the fabrication of fused silica microlens arrays (MLAs). Lasers can provide high temperatures at localized regions for a short pulse duration for surface patterning [11,12]. Fused silica [13] has a high melting temperature (1715 °C), a surface hardness of 6.0 Mohs, a spectral transmission range of 180 nm to 2 µm, and a very low coefficient of thermal expansion (CTE) of 0.54 × 10^−6^/°C. As the laser beam is very localized, low CTE can prevent cracks or fractures due to thermal gradient.

Due to the broad applications, several MLA fabrication techniques have been developed, such as lithography [14,15,16,17,18], surface tension-based curing [19,20], direct laser writing [21,22,23,24], thermal reflow [25,26,27,28], and embossing [29,30,31,32]. MLAs can be either planoconcave or planoconvex lenses. Concave MLAs have previously been obtained using femtosecond laser pulses on fused silica, followed by hydrofluoric acid etching [33,34], but this process takes 50 min to form concave lenses with a 30 µm aperture diameter. Convex MLAs are less commonly produced with laser techniques but have broader applications. Direct laser printing [35] can provide a convex lens with a 10 µm aperture diameter, but they still suffer from 60 min process times. Thermal reflow and micro nozzle printing [36,37] use photoresistors, UV-curable polymers, and other low-temperature materials to develop a convex lens. Template mold imprinting [38] uses masks and a ball end mill to form a lens that adds process steps, and the CMOS-compatible process [39] can generate high-quality microlenses but adds fabrication costs and process time.

In this paper, we describe a process that uses a picosecond laser for surface patterning and a CO_2_ laser to reflow the fused silica to form planoconvex MLAs. The described process eliminates masks and wet chemical etching. This fabrication process provides MLAs with high light transmission efficiency, cost-effectiveness, and process times as low as a few minutes per square inch area. We also fabricate and characterize the planoconcave lenses formed using individual CO_2_ and UV picosecond lasers and compare the results of surface morphology, surface profile, and beam characteristics with Zemax simulations. This process was used to fabricate planoconcave MLA on a sapphire (Al_2_O_3_) substrate using a 355 nm wavelength nanosecond pulsed laser. We discuss a few challenges in the CO_2_ laser melting of sapphire. We also compare laser-fabricated planoconvex MLAs with commercially available products.

The scientific studies have included MLAs fabricated using pulsed and CW CO_2_ lasers [40,41,42,43,44,45,46,47,48,49]. Yang et al. [28] used a picosecond laser and a CW CO_2_ laser with additional HF etching, and the paper focuses on the focal length study for only a planoconvex lens array. Sohn et al. [50] used a femtosecond pulsed laser and a CW CO_2_ laser, but the fabricated MLA suffered from very low transmission efficiencies. A single CO_2_ laser [51] was used in Q-switch and CW mode on the fabricated planoconvex lens but lacked data on transmission efficiencies. Similarly to other studies, Schwarz et al. [52] used a femtosecond laser and a CO_2_ laser to fabricate planoconvex lenses, but they also lack data on transmission efficiencies. Buttner et al. [53] used a pulsed laser and a CW CO_2_ laser, and the study focused on the radius of curvature dependence on laser fluence and electric field distribution, but no transmission data were reported. All the previous studies lack a comparative performance analysis between various pulsed and CW lasers. In our study, fabricating planoconcave microlenses by picosecond and CO_2_ lasers and combining picosecond micromachining with CO_2_ melting for planoconvex lenses allows us to perform a detailed performance comparison study of various types of lasers used for fabricating planoconvex and planoconcave microlenses. We also eliminate any chemical etching requirement. Table 1 compares various microlens results reported in the literature.

In this paper, we report results on the laser fabrication of planoconcave lenses by picosecond and CO_2_ lasers. Cylindrical lenses and planoconvex lenses were fabricated, first processing the fused silica with a picosecond laser and then melting it using a CO_2_ laser. Planoconcave MLA on sapphire was fabricated using a nanosecond laser. Finally, a comparative study was made.

## 2. Experimental

**Figure 1:**
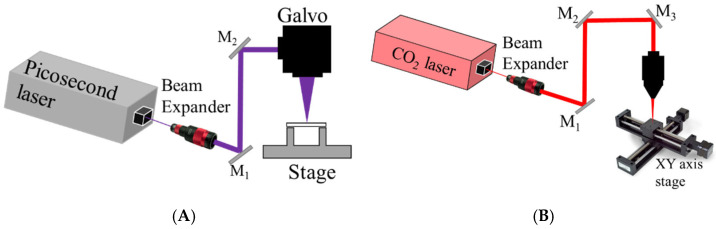
(**A**) Picosecond laser setup. (**B**) CO_2_ laser setup.

**Figure 2:**
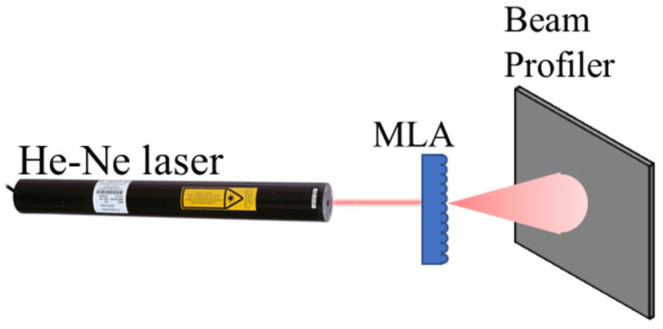
Beam profiler setup.

A 355 nm wavelength picosecond laser was used to inscribe arrays on fused silica and sapphire. The UV picosecond laser was a Spectra-Physics IceFyre 355-30, Milpitas, CA, USA, operated at a 350 kHz pulse repetition rate, 30 W average power, and 10 ps pulse width, shown in Figure 1A. The laser beam was directed using the Sino-Galvo SG7210 system, Zhenjiang, China. The sample inscribing velocity was set to 1000 mm/s. The CO_2_ laser was used to form planoconcave lens arrays and remelt fused silica for the planoconvex lens after the picosecond laser. The CO_2_ laser was a Senfeng SF1390i, Jinan, China, which is a continuous wave (CW) laser with 150 W average power and 300 µm spot size, as shown in Figure 1B. A Newmark e-track XY stage with 100 mm/s speed was used to form MLA on the fused silica. Fused silica was cleaned using DI water sonication. The Newport LBP2-HR-VIS2, Newport, RI, USA, beam profiler, with a 7.1 × 5.3 mm sensor size, S302C thermal power sensor, and 5 mW He-Ne, 632 nm wavelength laser, was used to characterize the transmission efficiency and beam profile as shown in Figure 2. An FEI Quanta 650 scanning electron microscope (SEM), Eindhoven, The Netherlands, was used to obtain surface morphology. Bruker atomic force microscopy (AFM), Billerica, MA, USA, and confocal microscopy were used to obtain surface roughness and morphology. Zemax software version R2 was used for ray tracing and beam profile simulations.

## 3. Results

### 3.1. Design of MLAs

**Figure 3:**
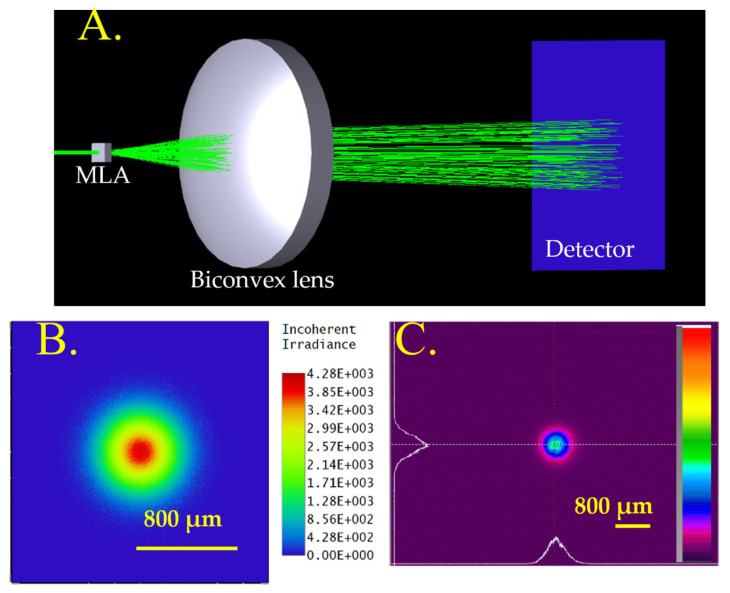
(**A**) Zemax Opticstudio simulation setup. (**B**) Simulated Gaussian beam profile. (**C**) Measured profile of He-Ne laser beam. Irradiance is in units of W/cm^2^.

The MLAs were designed by performing modeling using Zemax software in a non-sequential mode, which provides reflection ray and transmission ray data, as shown in Figure 3A. The lens data editor was used to define different parameters such as lens material, the radius of curvature, thickness, focal length, etc. Simulation provides a beam profile and a total irradiance plot at set positions in the ray path. It also provides a focal length of MLAs, and the simulation was performed using an 800 µm size Gaussian beam with a 632 nm wavelength laser beam passing through different MLAs. Figure 3B shows the beam profile of the simulated Gaussian beam, and Figure 3C shows the X and Y-measured profile of the typical He-Ne laser beam.

### 3.2. Concave Lens Fabrication on Fused Silica by CO_2_ Laser

**Figure 4:**
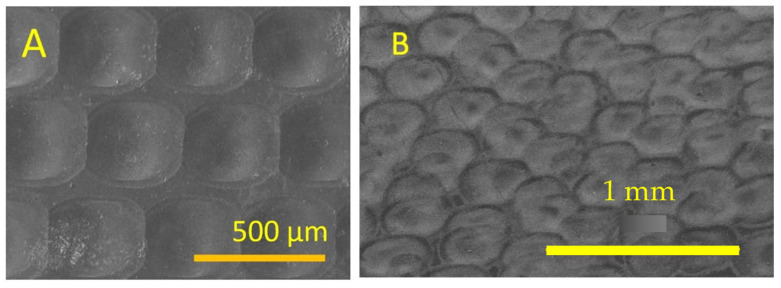
Concave lenses manufactured with CO_2_ laser. (**A**) Zero overlaps. (**B**) Random overlaps.

We fabricated concave MLAs on fused silica with CO_2_ laser irradiation. A 300 µm diameter lens shown in Figure 4A was fabricated using a laser power density of 43.6 kW/cm^2^ and an exposure time of 0.5 s. Due to the periodic nature and small diameter of the microlenses, optical diffraction rings were observed in the image of the transmitted beam. To eliminate diffraction effects, the microlenses were spaced randomly, as shown in Figure 4B. The CO_2_ laser-induced melting and solidification reshaped the surface into a concave form. The depth and radius of curvature of the formed concave lens depended upon laser fluence and irradiation time. An increase in time or irradiation fluence produced a higher curvature.

**Figure 5:**
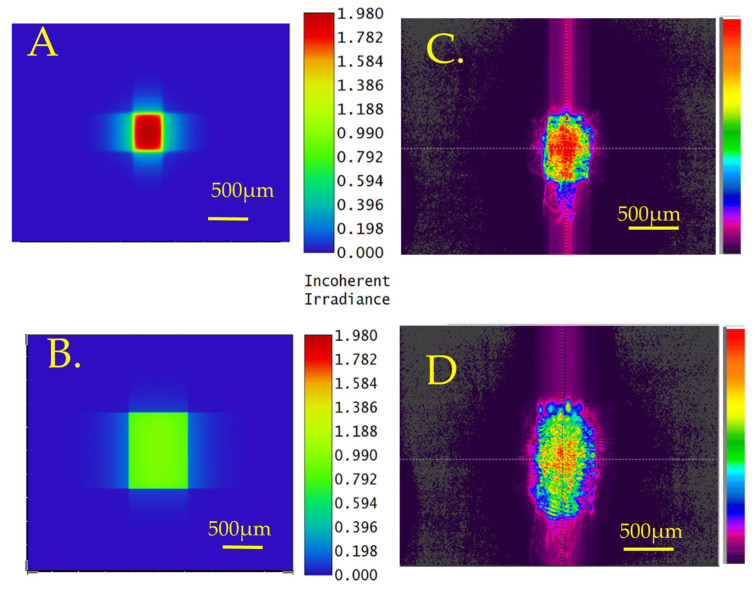
Simulated beam profile of a Gaussian beam after passing through the CO_2_-fabricated concave MLA at various distances from the lens: (**A**) 5 mm and (**B**) 10 mm. Experimentally measured beam profile at (**C**) 5 mm and (**D**) 10 mm.

The optical transmission efficiency for the CO_2_ laser-formed concave MLA was measured as 94.5% with an antireflection coating. The Zemax software simulations of the beam profile at 5 mm and 10 mm away from the lens are shown in Figure 5A,B, and the experimental results are shown in Figure 5C,D. As the Gaussian beam passes through the MLA, the beam expands and homogenizes. The measured divergence angle on the X and Y axes was 6.13° and 8.04°, respectively. The simulated divergence angle on the X and Y axes was 2.56° and 4.58°, respectively. The modeling results do not take into account the overlapping of lenses.

### 3.3. Concave Lens Fabrication on Fused Silica by Picosecond Laser

**Figure 6:**
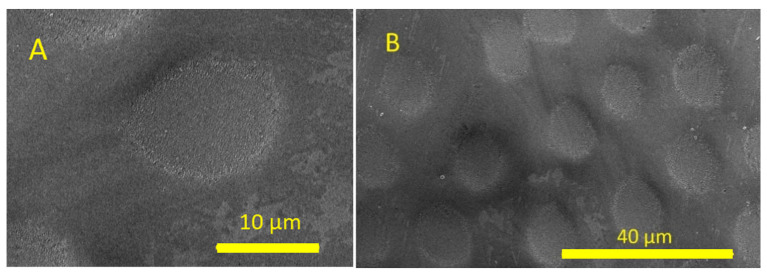
SEM images of concave MLAs on fused silica using a picosecond laser. (**A**) Individual concave lens. (**B**) Concave lenses with random spacing.

The concave MLA was fabricated using a picosecond laser. A 25 µm diameter lens was fabricated using a laser energy density of 4.66 J/cm^2^, as shown in Figure 6A. The lenses were spaced randomly to eliminate diffraction effects. The SEM image of these lenses is shown in Figure 6B.

**Figure 7:**
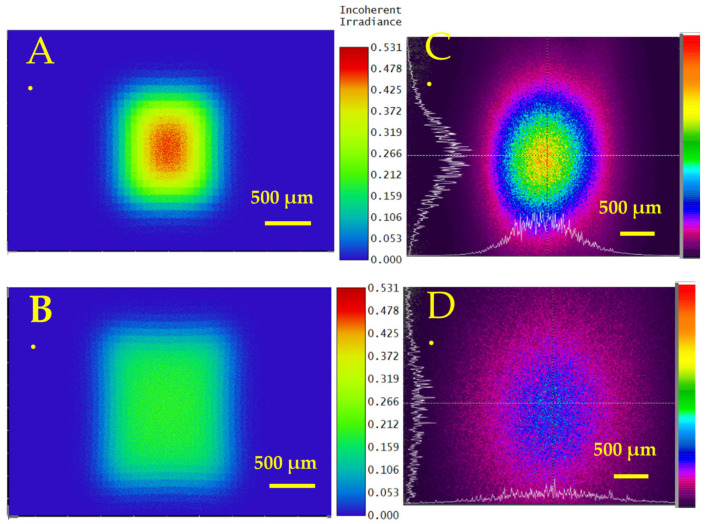
Simulated beam profile of Gaussian beam after passing through the picosecond laser-fabricated concave MLA at various distances from the lens: (**A**) 5 mm and (**B**) 10 mm. Experimentally measured beam profile after passing through the picosecond laser-fabricated concave MLA at various distances from the lens: (**C**) 5 mm and (**D**) 10 mm.

The optical transmission efficiency for picosecond laser-formed concave MLA was measured as 92.1% with an antireflection coating. The simulations of transmitted beam profiles at various distances from the lens of 5 mm and 10 mm are shown in Figure 7A,B, and the experimental results are shown in Figure 7C,D. The measured beam divergence angles on the X and Y axes were 11.99° and 15.09°, respectively. The simulated divergence angle on the X and Y axes was 8.56° and 11.4°, respectively.

### 3.4. Cylindrical Lens Fabrication on Fused Silica by Combining Picosecond and CO_2_ Lasers

**Figure 8:**
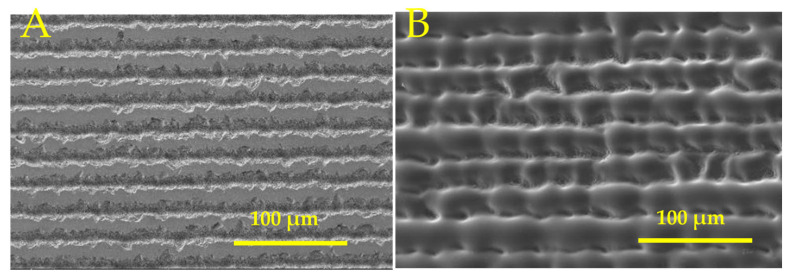
SEM image of (**A**) picosecond laser micromachined line pattern and (**B**) cylindrical lenses after CO_2_ laser melting.

The cylindrical lens was fabricated by inscribing periodic lines with 50 µm spacing to form vertical structures using the picosecond laser, as shown in Figure 8A. The vertical structures formed by the picosecond laser were remelted to form a cylindrical lens using the CO_2_ laser with a power density of 25.2 kW/cm^2^, as shown in Figure 8B.

**Figure 9:**
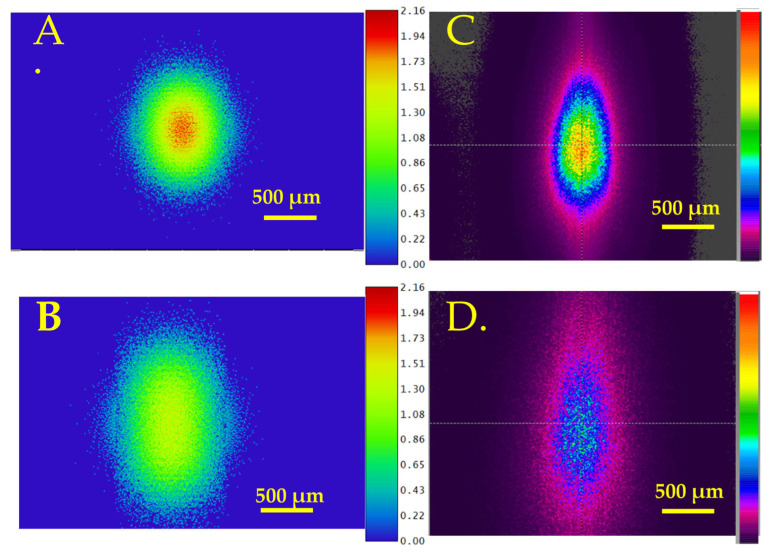
Simulated beam profile of a Gaussian beam after passing through the cylindrical MLA at various distances from the lens: (**A**) 5 mm and (**B**) 10 mm. Experimentally measured beam profile after passing through the cylindrical MLA at various distances from the lens: (**C**) 5 mm and (**D**) 10 mm.

The transmission efficiency for cylindrical MLA was measured as 86.7%. The simulations of the optical transmitted beam profile at various distances from the lens of 5 mm and 10 mm are shown in Figure 9A,B, and experimental measurements are shown in Figure 9C,D. The measured beam divergence angles on the X and Y axes were 5.55° and 11.33°, respectively. The simulated divergence angle on the X and Y axes was 2.86° and 11.4°, respectively.

### 3.5. Convex Lens Fabrication on Fused Silica by Combining CO_2_ and Picosecond Laser

**Figure 10:**
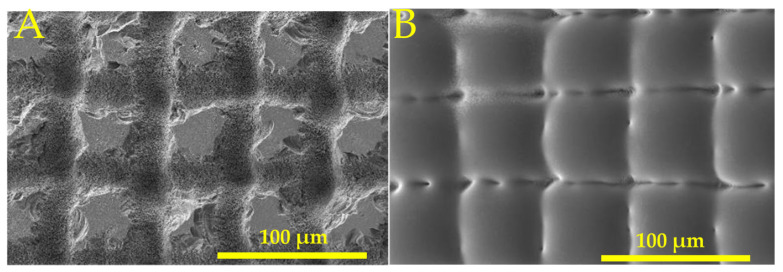
SEM image of (**A**) picosecond laser micromachined perpendicular line pattern and (**B**) convex lenses after CO_2_ laser melting.

The convex lens was fabricated by inscribing a perpendicular line array using a picosecond laser to form pillar structures, as shown in Figure 10A. The spacing between lines was 50 µm. The pillar structures were remelted using a CO_2_ laser with a power density of 25.2 kW/cm^2^ to form convex lenses. The SEM image of the convex MLA is shown in Figure 10B.

**Figure 11:**
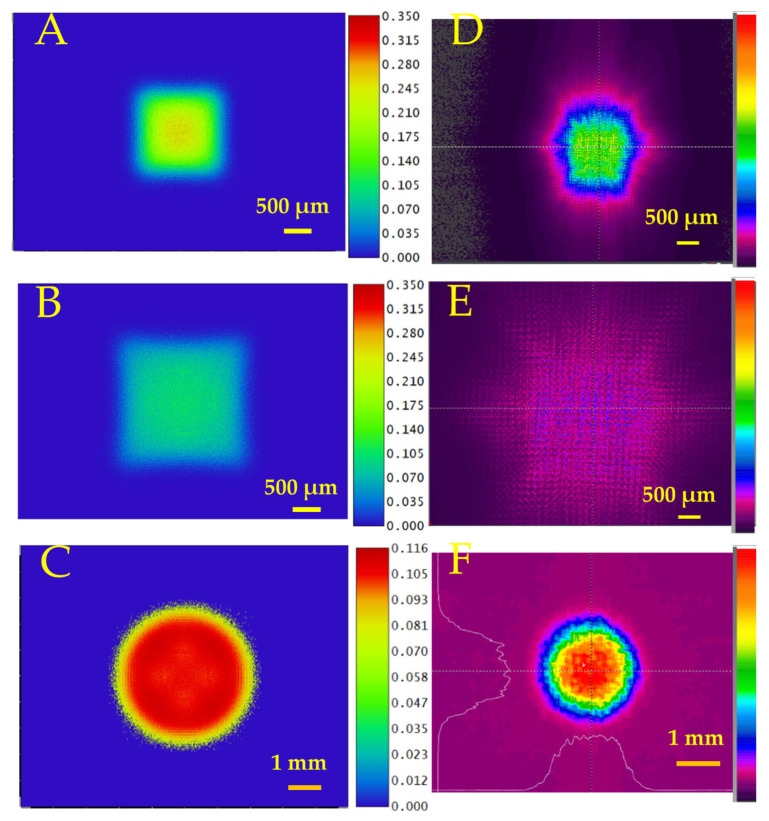
Simulated beam profile of Gaussian beam after passing through the convex MLA at various distances from the lens: (**A**) 5 mm, (**B**) 10 mm, and (**C**) flat-top beam profile after beam passes through MLA and through additional biconvex lens. Experimentally measured beam profile after passing through the convex MLA at various distances from the lens: (**D**) 5 mm, (**E**) 10 mm, and (**F**) flat-top beam profile after beam passes through MLA and through additional convex lens.

The measured transmission efficiency of convex MLA was 93.4% with an antireflection coating. The simulations of the beam profile after transmission through the lens at various distances from the lens of 5 mm and 10 mm are shown in Figure 11A,B. The experimentally measured beam profiles at various distances from the lens of 5 mm and 10 mm are shown in Figure 11D,E. The measured beam divergence angles on the X and Y axes were 13.35° and 15.56°, respectively. As the beam is passed through a convex lens, the beam profile transforms into a flat-top beam, as shown in Figure 11C,F. The simulated divergence angle on the X and Y axes was 8.56° and 10°, respectively.

**Figure 12:**
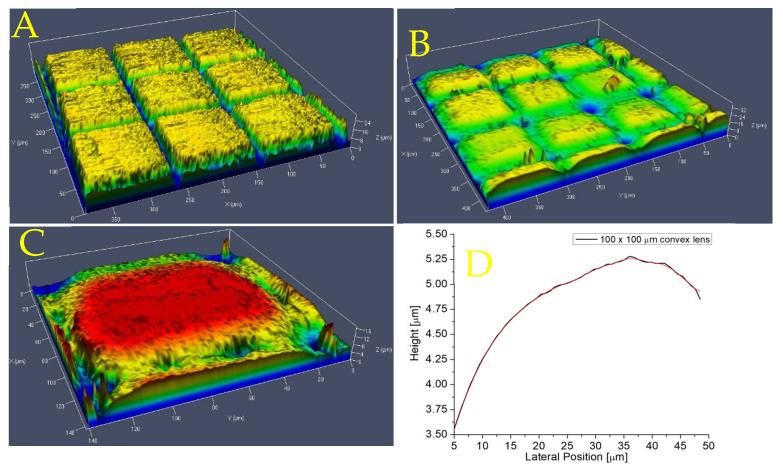
Surface profile of convex microlenses: (**A**) after UV picosecond machining, (**B**) after CO_2_ laser melting, (**C**) individual lens after CO_2_ laser melting, and (**D**) lens roughness measured by AFM.

The surface roughness of the microlens was measured using confocal microscopy and AFM after the picosecond laser machining and CO_2_ laser melting, and the results are shown in Figure 12A,B, respectively. Figure 12C shows the surface of a single microlens. Figure 12D shows the AFM data indicating a surface roughness of 75 nm, which is comparable to other studies on the laser fabrication of microlenses.

### 3.6. Concave Lens Fabrication on Sapphire by Picosecond Laser

**Figure 13:**
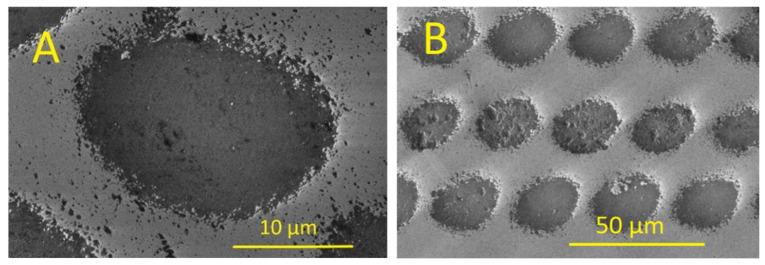
SEM image of concave MLAs on sapphire using a picosecond laser: (**A**) individual concave lens and (**B**) concave lens array.

The concave MLA was fabricated on sapphire using a picosecond pulsed laser. A 25 µm diameter lens was fabricated using a laser energy density of 4.66 J/cm^2^, as shown in Figure 13A. The lenses were spaced randomly to eliminate diffraction effects. The SEM image of these lenses is shown in Figure 13B.

**Figure 14:**
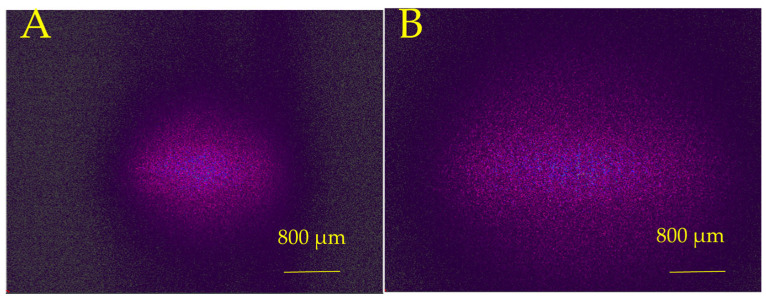
Experimentally measured beam profile after passing through the sapphire concave MLAs at (**A**) 5 mm and (**B**) 10 mm from the surface.

The optical transmission efficiency for picosecond laser-formed concave MLA on sapphire was measured as 81.99%. The experimental results of transmitted beam profiles at various distances from the lens of 5 mm and 10 mm are shown in Figure 14A,B. The measured beam divergence angles on the X and Y axes were 10.57° and 9.34°, respectively.

### 3.7. Commercially Available MLA

**Figure 15:**
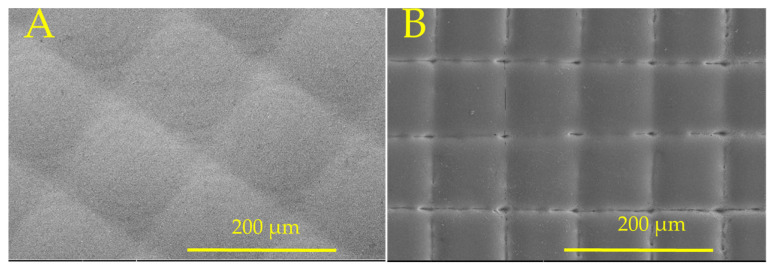
SEM image of (**A**) Thorlabs convex MLA and (**B**) fabricated 100 µm convex MLAs using picosecond and CO_2_ laser melting.

**Figure 16:**
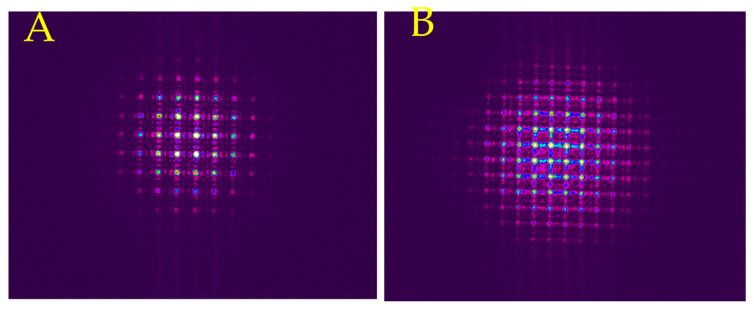
Experimentally measured beam profile after passing through the (**A**) Thorlabs convex Fresnel MLA at 5.2 mm and (**B**) fabricated 100 µm convex MLAs using picosecond and CO_2_ lasers at 7.5 mm.

We obtained the commercially available Thorlabs (MLA150-7AR) convex MLAs with 150 µm diameter microlenses with an anti-reflection coating (ARC). The SEM image of the Thorlabs MLA is shown in Figure 15A. The lens was a Fresnel microlens showing concentric rings. Its measured optical transmission efficiency was 91.8%. We fabricated 100 µm size convex lenses using a picosecond and CO_2_ laser, shown in Figure 15B. The measured beam profile for the Thorlabs MLA and the fabricated one are shown in Figure 16.

## 4. Discussion

**Figure 17:**
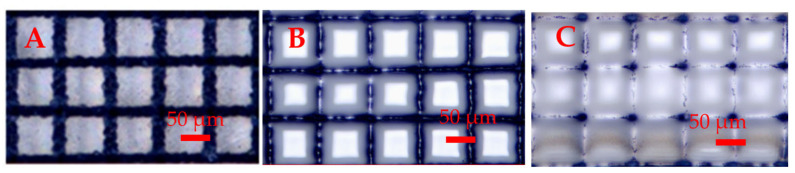
Optical images of convex microlenses: (**A**) fused silica machining with UV picosecond laser, (**B**) CO_2_ laser melting at 46 kW/cm^2^, and (**C**) CO_2_ laser melting at 61 kW/cm^2^.

The laser parameters, such as wavelength, power, pulse width, repetition rate, the overlap between laser scan lines, and scan speed, could be used to optimize the microlens performance. The fused silica is transparent in the visible and near-infrared wavelengths, so we have used an ultraviolet laser of 355 nm wavelength for our investigations. Laser wavelengths of 1030 and 1064 nm have been reported for the fabrication of microlenses, where absorption could occur through two-photon absorption. A laser pulse width of 10 picoseconds was used for the micromachining of lines to minimize any thermal damage to the fused silica, and melting was achieved by a CW CO_2_ laser. In the literature, the use of femtosecond, picosecond, and nanosecond lasers for microlens fabrication has been reported, and a shorter laser pulse width minimizes heat generation. For the melt reshaping of fused silica, a CO_2_ laser has been the most common tool. The CO_2_ laser power needs to be optimized, as too low a laser power will limit the melting depth and curvature, while too high a power will cause the spreading of the melt, as shown in Figure 17. Table 2 shows the optimized parameters used in this study. After CO_2_ laser melting at 25.2 kW/cm^2^, 46 kW/cm^2^, and 61 kW/cm^2^, we measured the focal lengths of 339.6 µm, 344.91 µm, and 425.1 µm, respectively.

We have shown that micro concave lens arrays can be fabricated using picosecond or CO_2_ lasers, and convex arrays can be fabricated by first processing fused silica with a picosecond laser, followed by melting with a CO_2_ laser. In this section, we discuss the MLA’s optical characteristics with simulated data and the influence of microlens shape on the laser beam profile as light passes through the MLAs. Figure 5 and Figure 7 show simulated and experimentally measured beam profiles from the CO_2_ laser and picosecond laser-fabricated concave MLAs, respectively. The measured beam shapes at different positions agree with the simulations. In Figure 5A,B, the simulations produce a rectangular beam; this is due to the ellipticity of the laser beam during fabrication, producing an elliptical concave shape. Even with random overlapping of the concave lens, we still observe some diffraction effects as large streaks in Figure 5C,D. The picosecond laser-focused beam spot size was 25 µm, which is significantly smaller than the 300 µm spot size of the CO_2_ laser. The measurements on picosecond MLAs using an 800 µm laser beam, when it passes through more MLA, diffraction effects were eliminated. The concave MLA produced by the picosecond laser has a larger curvature compared to the CO_2_ laser process, resulting in a smaller focal length and higher divergence angles.

Figure 9 and Figure 11 show simulated and experimentally measured beam profiles from the cylindrical and convex MLAs, respectively. The measured beam shapes at different positions agree with the simulations. The remelting process from the CO_2_ laser reduces surface roughness and increases optical transmission efficiency. From Figure 11F, we can change a Gaussian beam profile to a flattop beam.

The simulated beam divergence was lower than the measured beam divergence because we simulated using individual lenses rather than an array to reduce simulation times. Also, the simulations do not take surface roughness and other individual lens imperfections into account, which can increase beam divergence.

The sapphire MLAs, fabricated using a picosecond laser, had a transmission efficiency of 82% with no anti-reflection coating. The sapphire MLAs fabricated with the picosecond laser were shallower than fused silica lenses made with the same parameters. We have previously studied sapphire micromachining using a 355 nm nanosecond pulsed laser. The nanosecond pulse width 355 nm wavelength laser melted sapphire material instead of material ablation. This produced smooth concave features, as shown in Figure 13A. The sapphire has a higher refractive index than fused silica, so higher transmitted beam divergence was observed. To form convex lenses on sapphire, we produced similar patterns to fused silica, as shown in Figure 10A, and melted them using a CO_2_ laser. This process did not produce a convex lens, as the absorption coefficient for sapphire at 10.6 µm wavelength is ~2.5 × 10^2^ cm^−1^ compared to fused silica, which is ~1 × 10^3^ cm^−1^. This resulted in the CO_2_ laser being absorbed much deeper into the sapphire, resulting in cracking. To increase the absorption coefficient, the sapphire sample needs to be heated to more than 500 °C. The sapphire is transparent from UV to the mid-infrared wavelength of 5 microns, allowing the demonstration of microlenses for infrared applications.

To compare our process to commercially available MLAs, we created larger-sized convex MLAs similar to Thorlabs MLAs, shown in Figure 16A,B. From the beam profiles, we observed individual beam paths when measured at 5.2 mm and 7.5 mm from the surface of the Thorlabs MLA and laser-fabricated sample, respectively. We observe diffraction effects for both MLAs, but the fabricated sample has significantly higher diffracted light intensity. The diffraction effects can be minimized by eliminating any defects on the surface and randomizing the lenses.

This study details the rapid fabrication of large-area MLAs depending on the application and optimizes the resulting size of the lenses, focal lengths, and beam shape. Table 2 provides comparative data for the various types of MLA fabricated by the laser processing method. The transmission efficiency can be further improved by applying an antireflection coating (ARC) on MLA. Scattering effects can be eliminated by chemical etching to reduce surface roughness. The focal lengths of the MLA depend on the radius of curvature, which can be controlled by laser parameters such as laser fluence, scan speed, and repetition rate.

## 5. Conclusions

In this work, we report the successful fabrication of concave MLA by picosecond laser ablation and by CO_2_ laser and convex MLA by combining picosecond micromachining with a CO_2_ laser-induced melting process. The fabricated fused silica MLAs had a high light transmission efficiency of >94%. We simulated the transmitted light beam profile for the MLAs at different distances from the lens using Zemax software. A good agreement between the simulations and measurements was observed. We also fabricated planoconcave MLAs on sapphire. A comparative investigation was made between the various methods for MLA fabrication. The laser fabrication process is faster and can generate large-area MLAs. The laser process can be applied to other optical materials like sapphire, silicon, and zinc sulfide for the fabrication of microlenses.

## Figures and Tables

**Table 1:** Summary of published papers on laser microlens fabrication and measurements.

Paper	Type of Lens	Pulse Width, Wavelength	T	R (nm)	FL	Thermal Treatment
This study	Concave, Convex, and Cylindrical	355 nm ps, and 10.6 µm, CW	>94%	75	180 µm	No thermal treatment
[40]	Convex	1030 nm fs, 1064 nm ns, 10.6 µm, CW	-	3.90	1 mm	500 °C
[41]	Convex	1064 nm ns, 10.6 µm, CW	-	9.6	1 mm	500 °C
[42]	Cylindrical lenses	1030 nm fs, 10.6 µm, CW	-	-	18 µm	No thermal treatment
[43]	Convex and Cylindrical lenses	1030 nm fs, 10.6 µm, CW	-	783	125 µm	No thermal treatment
[44]	Cylindrical lenses	1030 nm fs, 10.6 µm, CW	-	10	125 µm	No thermal treatment
[45]	Concave lens	10.6 µm, CW	-	475	-	No thermal treatment
[46]	Concave lens	10.6 µm, CW	-	-	181 µm	Not provided
[47]	Axicon	1030 nm fs, 10.6 µm, CW	-	34	2 mm	No thermal treatment
[49]	Concave	10.6 µm, CW	-	78	-	No thermal treatment

Note: T—transmission, R—roughness, FL—focal length, ps—picosecond, fs—femtosecond, ns—nanosecond, CW—continuous wave.

**Table 2:** Comparison of lens parameters for various fabrication processes.

Parameter	ps Laser Fabrication (Concave Lens)	CO_2_ Laser Fabrication (Concave Lens)	ps + CO_2_ Laser Fabrication (Convex Lens)	ps + CO_2_ Laser Fabrication (Cylindrical Lens)
Wavelength	355 nm	10.6 µm	355 nm + 10.6 µm	355 nm + 10.6 µm
Pulse width	10 ps	CW	6 ps +CW	6 ps +CW
Power/energy density	4.7 J/cm^2^	43.6 kW/cm^2^	4.7 J/cm^2^ + 25.2 kW/cm^2^	4.7 J/cm^2^ + 25.2 kW/cm^2^
Repetition rate	350 kHz	Continuous	350 kHz + CW	350 kHz + CW
Scan speed (mm/s)	1000	100	1000 + 100	1000 + 100
µ-lens height (µm)	6.14	27.4	6.5	3.1
Simulated focal length (µm)	−262.5	−2251.6	156.5	34.8
Measured focal length (µm)	−145.64	−1796.7	177.49	55.69
Transmission efficiency (before ARC)	85.2%	90.8%	84.3%	86.7%
Transmission efficiency (after ARC)	92.1%	94.5%	93.4%	Not coated
Simulated beam divergence (degrees)	X: 8.56 Y: 11.4	X: 2.56 Y: 4.58	X: 8.56 Y: 10.00	X: 2.86 Y: 11.4
Measured beam divergence (degrees)	X: 11.99 Y: 15.09	X: 6.13 Y: 8.04	X: 13.35 Y: 15.56	X: 5.55 Y: 11.33
Fabrication time for 1-inch MLA	2 min	1 min	4 min	4 min

ARC, antireflection coating, ps, picosecond.

## Data Availability

Data underlying the results presented in this paper are not publicly available at this time but may be obtained from the authors upon reasonable request.
